# Editorial: Harnessing tumor microenvironment for gynecologic cancer therapy

**DOI:** 10.3389/fimmu.2024.1407128

**Published:** 2024-04-08

**Authors:** Linying Zhou, Ming Yi

**Affiliations:** ^1^ Department of Gynecology, Longquan People’s Hospital, Lishui, China; ^2^ The First Affiliated Hospital, College of Medicine, Zhejiang University, Hangzhou, China

**Keywords:** gynecologic cancer, tumor microenvironment, cancer immunotherapy, cervical cancer, ovarian cancer, uterine cancer

The intricate crosstalk between gynecologic cancers and host immunity has been extensively researched, revealing fascinating insights into tumor development. The tumor microenvironment (TME), comprising various non-tumor cells and soluble mediators, plays a pivotal role in supporting gynecologic cancer development (1, 2). Among these elements, tumor-infiltrating lymphocytes (TILs) emerge as defenders, equipped to identify and eliminate cancer cells. Additionally, the TME encompasses cancer-associated fibroblasts (CAFs), endothelial cells, chemokines, cytokines, growth factors, and antibodies, collectively regulating cancer initiation, advancement, and even treatment response (3–5). Cancer cells and other TME components release a multitude of immunomodulatory signals that can either inhibit or activate immune cell functions, effectively shaping the immune response (6–11). Consequently, the TME has the potential to switch the immune system from an anti-tumor mode to a pro-tumor state, depending on its composition (**Figure 1**). Encouragingly, therapeutic approaches targeting TME components, including myeloid-derived suppressor cells (MDSCs), tumor-associated macrophages (TAMs), and regulatory T cells (Tregs), and have demonstrated promising anti-tumor activity in both preclinical and clinical studies (12–18). Therefore, exploring the predictive and therapeutic values of the TME holds significant promise for advancing gynecologic cancer treatment. Here, we publish a Research Topic of six articles focusing on the TME-targeted treatment strategies for gynecologic cancers.

**Figure 1:**
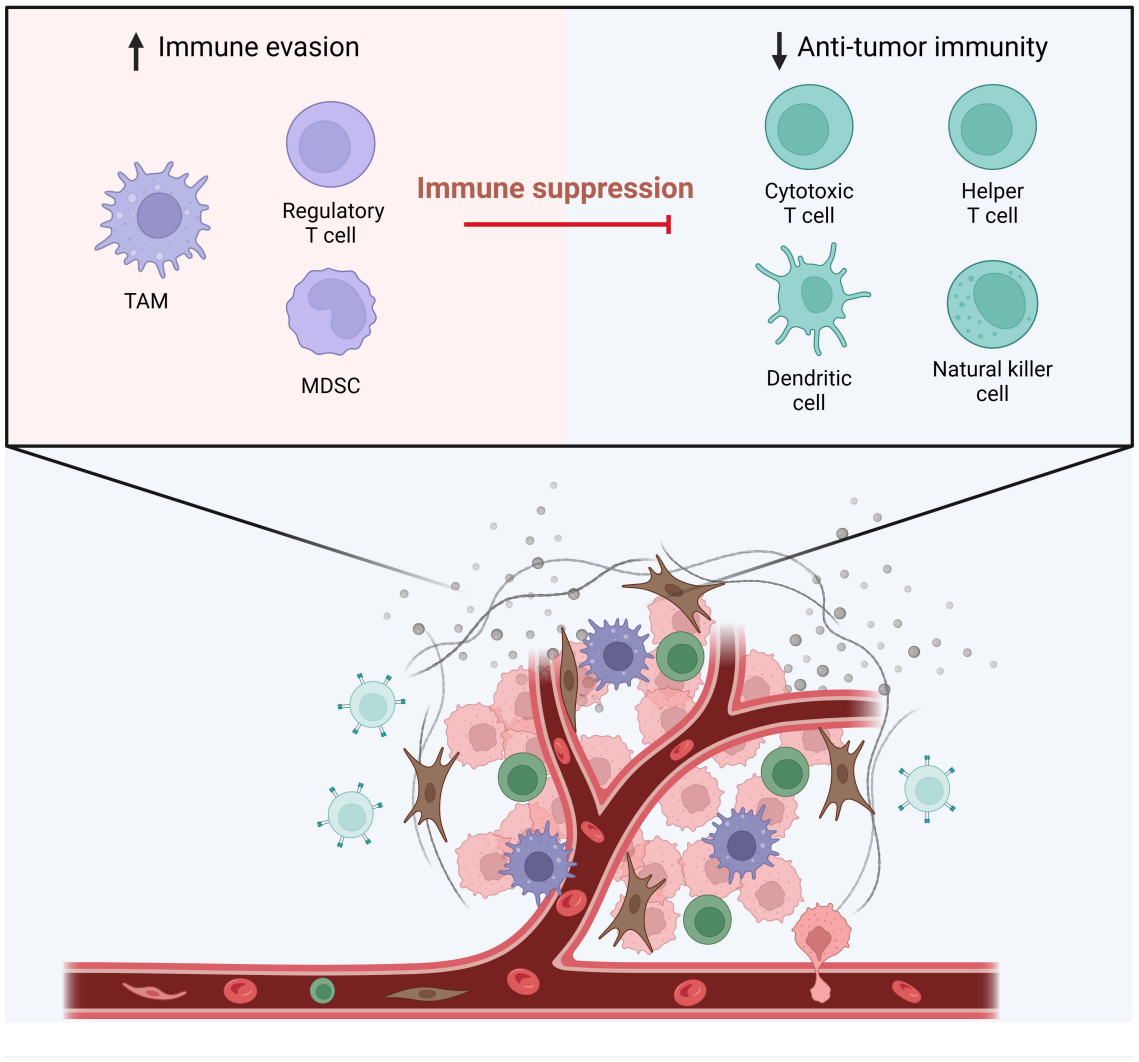
The Tumor Microenvironment and Immune Cell Dynamics. This figure illustrates the dynamic interactions between various immune cells within the tumor microenvironment. On the left, the components contributing to immune evasion are highlighted, featuring regulatory T cell (Treg), tumor-associated macrophages (TAM) and myeloid-derived suppressor cells (MDSCs) that play a role in immune suppression. On the right, the elements involved in anti-tumor immunity are depicted, which include cytotoxic T cells, helper T cells, dendritic cells, and natural killer cells. These cells are shown as active participants in the immune response against tumor cells. In the tumor microenvironment, the functions of these anti-tumor immune cells are hampered. (Adapted from “The Tumor Microenvironment: Overview of Cancer-Associated Changes”, by BioRender 2024).

The review by Yu et al. emphasizes the pivotal role of angiogenesis in the efficacy of cancer immunotherapy, particularly in the context of ovarian cancer. It outlines how angiogenesis, the formation of new blood vessels, not only supports tumor growth and metastasis but also significantly impacts the TME, thereby influencing the success of immunotherapies such as immune checkpoint inhibitors (ICIs). By fostering inadequate blood perfusion, hypoxia, and immune evasion through abnormal tumor vasculature, angiogenesis creates a challenging barrier to effective immunotherapy. Anti-angiogenesis therapy, exemplified by drugs like bevacizumab, targets these vascular abnormalities to not only disrupt tumor blood supply but also to potentially remodel the TME, thereby enhancing antitumor immunity. Clinical and preclinical studies have demonstrated the synergistic benefits of combining anti-angiogenesis therapy with ICIs, showing improved antitumor responses in ovarian cancer.

Besides, the review from Blanc-Durand et al. discusses the intricacies of ovarian cancer (OC) treatment, focusing on the TME and the challenges in harnessing ICIs for therapy. OC is characterized by an immunosuppressive TME that hinders the efficacy of these treatments (19). The complex interaction of tumor cells with various immune cells, including TILs, TAMs, and MDSCs, contributing to cancer immune escape. Although ICIs have not significantly improved patient outcomes in OC, the review highlights the ongoing efforts to understand and manipulate the TME for better therapeutic strategies. It mentions the potential of targeting TILs, exploiting the role of B-cells, and overcoming the immunosuppressive tactics of OC cells to develop more effective immunotherapies. The review also touches on the significance of novel biomarkers, adoptive cell therapy, vaccines, and next-generation ICIs, emphasizing the necessity of individualized treatment approaches to overcome the challenges presented by the OC TME.

Chen et al. comprehensively summarize the critical roles of Mucin 1 (MUC1) and Mucin 16 (MUC16) in cancer, highlighting their contributions to tumorigenesis, proliferation, metastasis, and immune modulation. MUC1 and MUC16, due to their overexpression and abnormal glycosylation in various cancers, become pivotal in mechanisms like cell proliferation, resistance to apoptosis, chemoresistance, and evasion of immune surveillance. Their involvement in modifying the TME and interaction with immune cells makes them promising targets for cancer therapeutics. The review delves into the molecular mechanisms by which MUC1 and MUC16 influence cancer progression, including their impact on cellular signal transduction, immune response regulation, and modulation of the tumor microenvironment. It also covers the latest advances in targeting these mucins through immunotherapies and targeted drugs, aiming to enhance the precision and efficacy of cancer treatments.

Li et al. elucidates the promising role of STING agonists, specifically MSA-2 (20), in enhancing the cervical cancer immune microenvironment and overcoming resistance to anti-PD-1 therapy. By leveraging data from TCGA and GEO datasets, the research demonstrates a positive correlation between the expression of STING downstream genes and improved prognosis in cervical cancer, alongside a notable enhancement in immune infiltration. Through scRNA-seq and *in vivo* murine models, MSA-2 was shown to activate key components within the TME, facilitating robust intercellular communication and intensifying immune cell interactions. The combination of MSA-2 with anti-PD-1 therapy significantly suppressed tumor growth compared to monotherapies, indicating a synergistic effect that boosts the antitumor immune response. This study underscores the transformative potential of combining STING agonists with immune checkpoint inhibitors, offering a novel therapeutic strategy to improve outcomes in cervical cancer treatment.

Moreover, Guo et al. analyze OC expression profiles to identify clinically relevant subtypes and develop a precise diagnostic model. Utilizing gene expression datasets from TCGA and GEO databases, the research classify OC into three robust subtypes based on survival-related genes, revealing significant differences in prognosis and molecular characteristics among the subtypes. Employing a machine learning algorithm, specifically the support vector machine (SVM), the researchers develop a diagnostic model that demonstrated superior performance in classifying OC subtypes, with impressive AUC values. This innovative approach not only offers new insights into the complex nature of OC but also provides a valuable diagnostic tool that can enhance clinical decision-making. Finally, the case report by Bian et al. on advanced cervical cancer with giant metastatic lymph node lesions demonstrates significant patient improvements and extended progression-free survival through a novel combination of local radiotherapy and immunotherapy, following conventional chemoradiotherapy failure. It suggests reevaluating the role of lymph nodes in tumor therapy, proposing that their integrity before immunotherapy could enhance treatment effectiveness, presenting a new approach to cancer radioimmunotherapy.

This Research Topic compiles cutting-edge research on the role of TME in gynecologic cancers, unveiling novel insights into angiogenesis, immune evasion, and the therapeutic targeting of mucins and STING pathways to improve patient outcomes. Through detailed analysis and innovative therapeutic strategies, these studies underscore the importance of a multifaceted approach to understanding and manipulating the TME, offering new horizons in the treatment of gynecologic cancers.

## Author contributions

LZ: Writing – original draft, Writing – review & editing. MY: Conceptualization, Writing – original draft, Writing – review & editing.
